# Seawater nasal wash to reduce symptom duration and viral load in COVID-19 and upper respiratory tract infections: a randomized controlled multicenter trial

**DOI:** 10.1007/s00405-024-08518-y

**Published:** 2024-02-20

**Authors:** Ludovic de Gabory, Sophie Vallet, Gaëlle Naelten, Chantal Raherison-Semjen

**Affiliations:** 1 Department of Otolaryngology (ENT) and Head & Neck Surgery, Bordeaux University Hospital, Bordeaux, France; 2 University of Bordeaux, 33000 Bordeaux, France; 3 Virology Unit, Brest University Hospital Centre, Brest, France; 4 Laboratoire de La Mer, Saint Malo, France; 5 Department of Pneumology, Centre Hospitalier Universitaire de Guadeloupe, Guadeloupe, France

**Keywords:** COVID-19, Irrigation, SARS-CoV-2 viral load, Seawater, Upper Respiratory Tract Infections

## Abstract

**Purpose:**

The objective was to assess the efficacy of seawater nasal wash on symptom duration, intranasal viral load, household transmission in COVID-19 and URTIs.

**Methods:**

This prospective, randomized, controlled, multicentric, parallel study included 355 mild/moderate COVID-19 and URTI adults with rhinologic symptoms ≤ 48h. Active group performed 4-daily nasal washes with undiluted isotonic seawater versus control group (without nasal wash). Symptoms were self-assessed daily using the WURSS-21 questionnaire for 3 weeks. Viral load was measured by RT-PCR on nasopharyngeal swabs collected on Day 0, Day 5, Day 14 and Day 21. Digital droplet PCR was additionally performed for SARS-CoV-2.

**Results:**

Overall COVID-19 subjects recovered earlier the ability to accomplish daily activities in the active group (– 1.6 day, p = 0.0487) with earlier improvement of taste (– 2 days, p = 0.0404). COVID-19 subjects with severe nasal symptoms at D0 showed the earliest resolution of anosmia (– 5.2 days, p = 0.0281), post-nasal drip (– 4.1 days, p = 0.0102), face pain/heaviness (– 4.5 days, p = 0.0078), headache (– 3.1 days, p = 0.0195), sore throat (– 3.3 days, p = 0.0319), dyspnea (– 3.1 days, p = 0.0195), chest congestion (– 2.8 days, p = 0.0386) and loss of appetite (– 4.5 days, p = 0.0186) with nasal wash. In URTIs subjects, an earlier resolution of rhinorrhea (– 3.5 days, p = 0.0370), post-nasal drip (– 3.7 days, p = 0.0378), and overall sickness (– 4.3 days, p = 0.0248) was reported with nasal wash.

Evolution towards more severe COVID-19 was lower in active vs control, with earlier viral load reduction in youngest subjects (≥ 1.5log10 copies/10000 cells at Day 5: 88.9% vs 62.5%, p = 0.0456). In the active group, a lower percentage of SARS-CoV-2 positive household contacts (0–10.7%) was reported vs controls (3.2–16.1%) among subjects with Delta variant (p = 0.0413).

**Conclusion:**

This trial showed the efficacy and safety of seawater nasal wash in COVID-19 and URTIs.

**Trial registration:**

Trial registry ClinicalTrials.gov: NCT04916639. Registration date: 04.06.2021.

**Supplementary Information:**

The online version contains supplementary material available at 10.1007/s00405-024-08518-y.

## Introduction

Upper Respiratory Tract Infections (URTI) are the most frequent sources of morbidity with 2–5 colds/adult/year and 7–10 colds/infant/year [1, 2]. The emergence of SARS-CoV-2 and its predominant mild to moderate COVID-19 forms, expanded the occurrence of upper respiratory symptoms. As with common cold, flu and the usual URTIs, mild or moderate COVID-19 cases do not require hospitalization or advanced medical care. Likewise, the primary mode of transmission of SARS-CoV-2 and URTI viruses is through respiratory droplets [3, 4]. Nasal irrigation is commonly prescribed for the prevention and treatment of URTI symptoms and other sinonasal conditions [5–10]. It provides mechanical cleaning of nasal content [11], with rare adverse events [12, 13]. It enhances mucociliary clearance thereby reducing the contact time of airborne elements with mucus [11, 14–16].

Nasal irrigation solutions close to seawater, i.e. slightly alkaline pH with richer content in magnesium, potassium, calcium and lower sodium chloride concentrations than saline solution have been found to be effective in improving nasal symptoms in children and adults [11]. They provide further improvement of nasal mucosa functionality, avoid impairment of ciliary beat frequency and healing process and optimize the clinical outcomes [13, 17]. Moreover, computational fluid dynamic simulations showed that continuous spray allows adequate physical parameters to remove nasal content and seemed appropriate for short-term diseases and prevention [18].

Based on the available published data in 2020 [19], the French Society of Otorhinolaryngology, published guidelines in favor of nasal wash to reduce COVID-19 symptoms [20–22]. Recent studies suggest that washes with isotonic saline solutions can reduce several upper respiratory tract symptoms of subjects infected with SARS-CoV-2 [23], reduce their hospitalization [24], and decrease their viral load [25]. Another study with a hypertonic solution also suggests washes can reduce the duration of URTIs, the transmission within household contacts and viral shedding [26]. These trials were performed with high-risk patients, a small number of subjects, in a hospital setting, in severe COVID-19 pneumonia, or with an acute design, that may limit their transposition to the general population. Further studies on a larger number of subjects and with a setting close to real-life are needed, including simultaneously follow-up of COVID-19 and URTIs subjects, and treatment with mineral-rich nasal irrigation solutions. Acceptability and at-home ease of use of the device are important factors in promoting patients’ compliance to nasal wash [27]. Clinical trials should therefore evaluate in a real-life setting, the effectiveness and safety of nasal wash devices commonly used in general population and whose reluctance to regular use is weak. The most frequently recommended and used nasal wash devices to treat symptoms of upper respiratory tract infections are isotonic nasal sprays [27, 28]. Unlike large-volume irrigation devices, mainly delivering normal saline (0.9% NaCl) or home-made solutions, nasal sprays provide solutions of varying mineral content, the richest being undiluted seawater [11]. Although normal saline was used as a reference solution, seawater nowadays appears to have superior properties. The most recent guidelines recommend using a comfortable nasal wash method with solutions close to seawater [29, 30].

The aim of our study was therefore to assess the efficacy of an isotonic undiluted seawater nasal spray to relieve COVID-19 and URTIs nasal symptoms, reduce intranasal viral load and virus transmissibility in subjects with mild to moderate COVID-19 disease and URTIs in a real-life setting.

## Materials and methods

### Trial design

This was a prospective, open-label, randomized (1:1), controlled, multicenter, parallel clinical trial. The study took place at 15 sites Medical Analysis Laboratories distributed across France between July 2021 and March 2022.

Eligible participants included adults (≥ 18 years) who spontaneously attended study sites for SARS-CoV-2-testing and presented self-reported nasal obstruction and/or rhinorrhea for up to 48h. Only patient with mild to moderate grade of infections were included [31]. Subjects required to take regular medications administered by nasal route or performed a nasal wash in the previous days were excluded from the study. No restrictions were added on the intake of other concomitant treatments (see Supplementary Table 1 for the full list of inclusion and exclusion criteria).

Subjects were randomized (unblinded) to nasal wash (active group) or no wash (control group). No placebo could be used due to its obvious nasal cleansing mechanical action.

The nasal irrigations were performed with the Physiomer® Normal Jet (Laboratoire de la Mer, Saint Malo, France), a nasal wash spray widely used in Europe for the relief of rhinologic symptoms and whose tolerance and acceptability have already been validated [11, 17, 32]. This medical device CE class IIa is a nasal spray delivering a continuous flow (2.5–4 ml/s) of sterile isotonic undiluted electrodialyzed seawater. Its slightly alkaline pH and mineral-rich composition have been already described [11]. Subjects were asked to wash each nostril 4 times/day (morning, midday, afternoon, and evening) for 3 weeks.

The 21 days follow-up included: 1) Enrolment/randomization visit on site (D0) involving a rapid SARS-COV-2 antigenic test (PanBio™ COVID-19 Ag Abbott Rapid Diagnostics) and questionnaire for baseline symptoms. A RT-PCR test (nasopharyngeal swab) was performed to confirm the indication and cycle threshold (Ct) baseline values (PerkinElmer® SARS-CoV-2 Real-time RT-PCR Assay); 2) D3, D5, D14 and D21 home visits by a nurse to perform nasopharyngeal swab for SARS-CoV-2 and URTI viral load and check on symptoms; 3) Daily online self-reported questionnaires for symptoms (WURSS-21 modified for COVID-19 symptoms including smell and taste disorders), medication and tolerance from D0 to D21.

Viral load assessments for SARS-CoV-2 and URTIs were analyzed in a central laboratory for RT-PCR quantification in Ct values (targeted SARS-CoV-2 N-gene and RdRp-gene), with further droplet digital PCR performed for SARS-CoV-2 (Bio-Rad® SARS-CoV-2 ddPCR kit 10000128901 revA) expressed in Log copies/10,000 cells. Analysis of URTI viruses were performed with multiplex RT-PCR (Respiratory Multi Well System r-gene®).

### Ethical approval and trial registration

The study was approved by Independent Ethics Committee and conducted in compliance with the Declaration of Helsinki (1964), the French Law L.1123-6 and guidelines for good clinical practice (NF EN ISO 14155 July 2020).

All patients signed a written informed consent prior to inclusion in the study. The study was registered at Clinicaltrials.gov in June 2021 with number: NCT04916639.

### Objectives and endpoints

The primary objective was to assess the efficacy of Physiomer® Normal Jet to reduce the duration of nasal symptoms (rhinorrhea and congestion) in subjects with COVID-19 and URTIs.

The primary endpoint was the number of days until the first follow-up wherein nasal symptoms resolved. This means “0” score on nasal congestion or rhinorrhea based on the Wisconsin Upper Respiratory Symptom Survey (WURSS-21) self-questionnaire, modified for COVID-19 symptoms [19, 33].

The secondary endpoints included: (1) the number of days until resolution of COVID-19 and URTIs-induced individual symptoms; (2) the percentage of subjects with evolution towards more severe COVID-19 stage; (3) the percentage of subjects with daily symptom relief; (4) the device-related symptoms relief; (5) the change from baseline in SARS-CoV-2 and URTI viral load in nasal cavities (6) the percentage of subjects with positive SARS-CoV-2 test among household contacts; (7) the tolerance to nasal wash; (8) the adverse events reported by subjects.

### Statistical methods

A sample size of 370 subjects was calculated using a type I error of 5% and power of 80% to detect duration of symptoms of 9.5 ± 3 days for the active group and 10.5 ± 3 days for the controls, plus a 20% rate of missing data, a 5% rate of severe cases and a 20% rate of false positives.

Statistical analyses were performed using R software version 4.0.3. The normal distribution was verified using the Shapiro–Wilk test. Quantitative variables were described as means with standard deviation, while qualitative variables were expressed as percentages. Significant differences between groups were determined using the Student t-test or the Wilcoxon Mann–Whitney U test for quantitative variables. The chi-square test or the Fisher-exact test were used to determine significant difference between qualitative variables. For the time course of symptom intensity, AUCs were calculated for each subject and each time interval to test the effect of time and randomization with a mixed model. For the percentage of SARS-CoV-2 positive household contacts, a Poisson regression model with mixed effects was carried out to take into account the non-independence of values for the same household during the study. For all tests, p < 0.05 was considered statistically significant. In case of lost to follow-up, missing data were not replaced. Subgroup analyses were performed on subjects with most severe nasal congestion and rhinorrhea at baseline (symptom score > 5 on the WURSS-21 scale, with 0 = Do not have symptom, 1 = Very mild, 3 = Mild, 5 = Moderate, 7 = Severe symptom) [33].

## Results

### Study populations and baseline characteristics

**Fig. 1:**
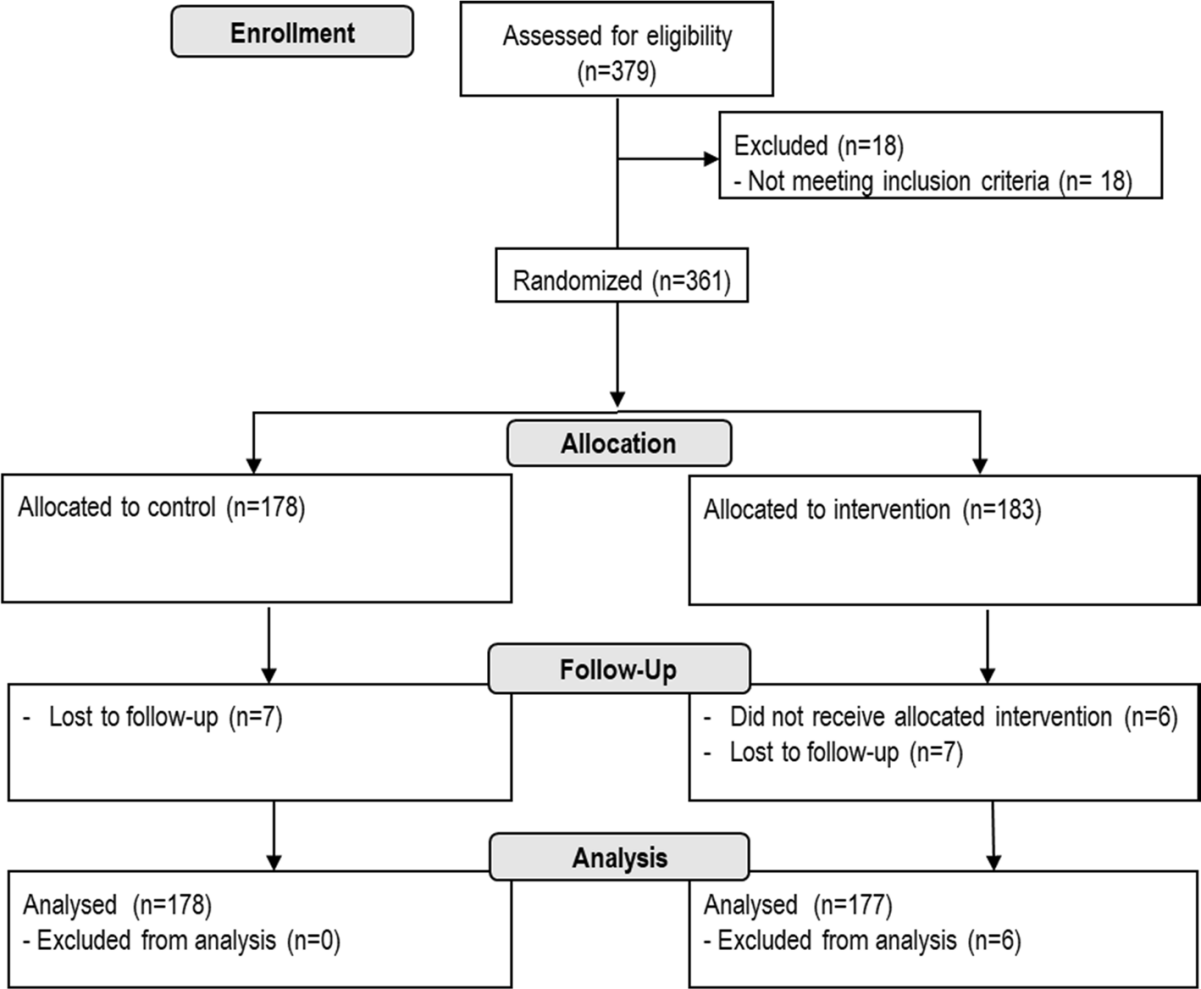
Consort diagram of the progress through the phases of the study

Among the 379 screened subjects, 355 were randomized to receive nasal wash (active group, n = 177) or not (control group, n = 178) (Fig. 1).

Following virological analysis: 56% (n = 199) of patients tested positive for SARS-CoV-2, 173 at Day 0 (COVID-19 population) and 26 from Day 3, 10.4% (n = 37) had URTI viruses, and 33.5% (n = 119) hadn’t identified virus despite clear common cold symptoms. Among COVID-19 population, 48.3% were classified as “mild” grade and 51.7% as “moderate”, with 29.5% of subjects vaccinated against SARS-CoV-2 (Table 1). Beyond rhinorrhea and nasal congestion, the most frequent COVID-19 reported symptom was fatigue (Supplementary Fig. 1). In accordance with public health recommendations [34], the analysis for SARS-CoV-2 variant types was systematically performed in daily practice during PCR analyses at the beginning of the study but this was no longer the case by the end of the study. Thus, 106 (61.3%) patients had variant screening: 53.8% of Omicron, 38.7% of Delta and 7.5% of alpha/wildtype.

**Table 1:** Baseline characteristics

	All symptomatic subjects			COVID-19 subjects			URTIs subjects		
	Control	Active	p-value	Control	Active	p-value	Control	Active	p-value
	N = 178	N = 177		N = 91	N = 82		N = 11	N = 26	
Age, mean (SD)	32.3 ± 10.4	31.2 ± 10.7	0.239	33.0 ± 9.8	32.8 ± 11.2	0.597	23.4 ± 6.6	27.8 ± 9.0	0.107
Gender									
Female, n (%)	122 (68.5)	120 (67.8)	0.971	61 (67.0)	56 (68.3)	0.988	8 (72.7)	16 (61.5)	0.710
Male, n (%)	56 (31.5)	57 (32.2)		30 (33.0)	26 (31.7)		3 (27.3)	10 (38.5)	
BMI, mean (SD)	24.3 ± 3.8	23.8 ± 3.4	0.512	24.0 ± 3.7	23.9 ± 3.5	0.934	21.3 ± 2.0	22.7 ± 2.8	0.172
Smoker, n (%)	9 (5.1)	9 (5.1)	n.a	3 (3.3)	3 (3.7)	n.a	0 (0)	3 (11.5)	n.a
Availability of SARS-CoV-2 vaccination status, n(%)	102 (57.3)	104 (58.8)	n.a	32 (35.2)	25 (30.5)	n.a	11 (100)	22 (84.6)	n.a
Fully SARS-CoV-2 vaccinated, n (%)	92 (90.2)	94 (90.4)	n.a	31 (96.9)	20 (80.0)	n.a	11 (100)	21 (95.5)	n.a

*n.a* no applicable

URTIs viruses detected from nasal swabs were: Human rhinoviruses or other Human enteroviruses (37.8%), Influenza virus A (27.0%), Human Coronavirus (16.2%), Human Adenovirus (13.5%), Human Bocavirus (2.7%) and Human respiratory syncytial virus (10.8%).

There were no significant differences regarding baseline data between randomization groups in the population as a whole, in COVID-19 and URTIs subsets (Table 1).

During the study period, 37.5% (n = 133) of subjects took symptomatic treatment. Twelve subjects used a treatment for nasal symptoms (3.38%) with close repartition between control and active groups: nasal decongestant (6/178, 3.37% in controls; 1/177, 0.56% in active group), oral decongestants (2/178, 1.12% in controls; 2/177, 1.13% in active group). Only one subject in the control group used nasal wash during the study.

### Efficacy on the resolution of rhinorrhea and nasal congestion

For the entire study population, the mean time to resolution of rhinorrhea and nasal congestion was similar for controls (6.6 ± 4.7, CI_95%_ [5.8; 7.4]), and for the active group (7.0 ± 4.9, CI_95%_ [6.3; 7.8]) (p = 0.7603).

Time to resolution in COVID-19 subjects was also similar for controls (6.4 ± 4.4, CI95% [5.5; 7.4]), and for the active group (6.4 ± 4.4 (CI95% [5.4; 7.5]) (p = 0.5050). Interestingly, the COVID-19 subjects with severe baseline nasal congestion and rhinorrhea, benefited from a shortening of nasal symptoms (– 2.1 days and – 1.7 days) with nasal wash, while non reaching statistical significance (p = 0.0778 and p = 0.2025 respectively).

In the same way, in URTIs population, a 2-days shorter while non-significant duration of nasal symptoms was reported in the active group (p = 0.1439). In URTIs subjects without concomitant treatment, the effect of nasal wash was more visible with a 4.2-days significantly earlier resolution of nasal symptoms (p = 0.0450).

### Efficacy on the resolution of other upper respiratory tract related symptoms

Regardless the etiology, the comparison between control and active group showed a difference that was all the more significant as the nasal symptoms were severe at baseline (Table 2).

**Table 2:** Days until symptom resolution

	All subjects				Subjects with severe rhinorrhea				Subjects with severe nasal congestion			
	Control	Active	p-value	Δ days	Control	Active	p-value	Δ days	Control	Active	p-value	Δ days
*All symptomatic subjects*	(n = 178)	(n = 177)			(n = 37)	(n = 29)			(n = 41)	(n = 28)		
Alteration/loss of smell	7.4 ± 5.7	5.4 ± 5.1	0.084	– 2	4.4 ± 3.1	5.9 ± 6.6	0.500	1.5	7.5 ± 5.6	3.6 ± 1.7	0.028*	**– 3.9**
Alteration/loss of taste	7.7 ± 5.8	4.7 ± 5.1	0.030*	**– 3**	10.0 ± 6.9	6.0 ± 6.7	–	– 4.0	6.1 ± 4.2	2.0 ± 0.0	–	– 4.1
Post-nasal drip	6.3 ± 5.0	5.9 ± 5.2	0.197	– 0.4	7.2 ± 5.3	4.3 ± 4.2	0.012*	**– 2.9**	7.1 ± 5.1	4.4 ± 2.4	0.037*	**– 2.7**
Face pain/heaviness	5.0 ± 3.8	5.1 ± 5.1	0.122	0.1	6.2 ± 4.7	3.6 ± 2.6	0.032*	**– 2.6**	6.0 ± 3.5	4.9 ± 4.1	0.084	– 1.1
Headache/migraine	5.2 ± 4.2	5.2 ± 4.9	0.241	0	6.0 ± 4.4	4.3 ± 3.8	0.075	– 1.7	6.5 ± 5.0	4.8 ± 4.8	0.035*	**– 1.7**
Chest congestion	4.5 ± 4.4	3.4 ± 3.1	0.140	– 1.1	4.4 ± 3.9	4.2 ± 3.6	0.555	– 0.2	5.2 ± 4.6	2.9 ± 1.4	0.049*	**– 2.3**
Shortness of breath/dyspnea	4.6 ± 3.9	4.0 ± 3.8	0.032*	**– 0.6**	4.5 ± 3.2	4.9 ± 4.9	0.250	0.4	5.5 ± 4.3	4.2 ± 4.3	0.049*	**– 1.3**
Loss of appetite	5.0 ± 4.5	4.1 ± 3.7	0.198	– 0.9	4.9 ± 3.7	2.4 ± 2.4	0.028*	**– 2.5**	5.3 ± 3.9	3.4 ± 5.2	0.024*	**– 1.9**
*COVID-19 subjects*	(n = 91)	(n = 82)			(n = 21)	(n = 14)			(n = 26)	(n = 16)		
Alteration/loss of smell	9.5 ± 5.7	6.7 ± 5.2	0.100	– 2.8	5.6 ± 2.9	6.0 ± 3.5	NA	0.4	8.5 ± 5.9	3.3 ± 1.2	0.028*	**– 5.2**
Alteration/loss of taste	9.0 ± 5.1	6.7 ± 5.6	0.147	– 2.3	6.7 ± 2.1	6.0 ± 5.7	NA	– 0.7	6.9 ± 4.0	2.0 ± 0.0	–	– 4.9
Post-nasal drip	6.5 ± 4.6	5.6 ± 5.0	0.077	– 0.9	7.7 ± 4.6	3.6 ± 2.6	0.010*	**– 4.1**	7.0 ± 3.8	4.5 ± 2.7	0.037*	**– 2.5**
Face pain/Heaviness	5.6 ± 3.9	5.0 ± 4.7	0.077	– 0.6	8.1 ± 4.6	3.6 ± 2.2	0.007**	**– 4.5**	7.3 ± 3.5	3.9 ± 1.8	0.005**	**– 3.4**
Sore throat	5.5 ± 4.6	5.6 ± 5.0	0.384	0.1	6.4 ± 4.8	3.1 ± 2.0	0.031*	**– 3.3**	6.6 ± 4.8	5.9 ± 4.5	0.296	– 0.7
Chest congestion	5.1 ± 4.6	3.8 ± 3.5	0.132	– 1.3	5.6 ± 4.0	3.0 ± 2.1	0.083	– 2.6	5.9 ± 5.0	3.1 ± 1.3	0.038*	**– 2.8**
Dyspnea	4.7 ± 4.2	3.5 ± 2.8	0.078	– 1.2	5.1 ± 3.6	3.6 ± 3.4	0.076	– 1.5	6.0 ± 5.0	2.9 ± 1.5	0.019*	**– 3.1**
Headache/migraine	6.0 ± 4.5	5.8 ± 5.0	0.222	– 0.2	6.9 ± 4.2	4.7 ± 3.6	0.103	– 2.2	7.4 ± 5.6	4.3 ± 4.2	0.022*	**– 3.1**
Loss of appetite	5.7 ± 4.4	4.9 ± 3.8	0.330	– 0.8	6.5 ± 3.9	2.0 ± 1.7	0.018*	**– 4.5**	7.0 ± 3.8	1.5 ± 1.0	–	– 5.5
Accomplish daily activities	6.6 ± 4.8	5.0 ± 4.1	0.048*	**– 1.6**	6.5 ± 4.8	3.8 ± 3.2	0.071	– 2.7	8.3 ± 5.5	3.7 ± 2.7	0.011*	**– 4.6**
*URTI subjects*	(n = 11)	(n = 26)			(n = 7)	(n = 14)			(n = 7)	(n = 11)		
Rhinorrhea	12.2 ± 5.8	8.7 ± 4.3	0.037*	**– 3.5**	13.4 ± 6.3	11.0 ± 4.3	0.179	– 2.4	12.0 ± 5.4	10.1 ± 4.9	0.246	– 1.9
Plugged nose/congestion	11.8 ± 7.2	9.0 ± 5.6	0.136	– 2.8	14.0 ± 6.6	10.4 ± 4.9	0.096	– 3.6	11.4 ± 6.9	9.8 ± 4.3	0.283	– 1.6
Post-nasal drip	7.7 ± 6.3	4.0 ± 3.4	0.037*	**– 3.7**	8.3 ± 6.6	2.4 ± 1.5	0.040*	**– 5.9**	9.0 ± 7.2	2.4 ± 1.1	0.054	– 6.6
Cough/dry cough	10.2 ± 7.9	6.1 ± 5.2	0.113	– 4.1	13.8 ± 7.6	5.4 ± 6.0	0.014*	**– 8.4**	13.5 ± 8.2	6.7 ± 5.9	0.063	– 6.8
Overall sickness	12.9 ± 6.3	8.6 ± 4.7	0.024*	**– 4.3**	14.1 ± 5.6	10.7 ± 5.5	0.112	– 3.4	13.9 ± 5.3	9.6 ± 5.8	0.081	– 4.3

*significative p values

For all subjects, whatever the baseline degree of symptom severity, we observed a significantly earlier resolution of taste disorders (– 3 days, p = 0.0305) and shortness of breath/dyspnea (– 0.6 day, p = 0.0323) (Table 2). Considering the days until first symptom reduction, we showed a significantly earlier improvement of olfactory (– 2.2 days, p = 0.0170) and taste alterations (– 2.1 days, p = 0.0071) in favor of active group.

Patients with severe rhinorrhea showed a significantly earlier resolution of post-nasal drip (– 2.9 days, p = 0.0125), face pain/heaviness (– 2.6 days, p = 0.0323) and loss of appetite (– 2.5 days, p = 0.0288) in the active group vs controls.

Subjects with severe nasal congestion using nasal washes showed the significantly earliest resolution of olfactory disorders (– 3.9 days, p = 0.0281), with significant resolution of post-nasal drip (– 2.7 days, p = 0.0374), headache (– 1.7 days, p = 0.0351) and loss of appetite (– 1.9 days, p = 0.0244), compared to controls (Table 2).

#### COVID-19 subjects

In all COVID-19 subjects, there was a trend for earlier resolution of symptoms with nasal washes but without statistically significant difference except for the ability to accomplish daily activities (– 1.6 days, p = 0.0487) (Table 2). Considering the days until first symptom reduction, taste disorders started to improve 2 days significantly earlier in active group (p = 0.0404).

The benefit of nasal irrigation was more visible for patients with severe baseline symptoms such as subjects suffering from severe rhinorrhea who showed the earliest resolution of post-nasal drip (– 4.1 days, p = 0.0102), face pain/heaviness (– 4.5 days, p = 0.0078), sore throat (– 3.3 days, p = 0.0319) and loss of appetite (– 4.5 days, p = 0.0186) with nasal wash (Table 2).

Similarly, subjects with severe nasal congestion using nasal washes showed significantly earlier resolution of post-nasal drip (– 2.5 days, p = 0.0373), face pain/heaviness (– 3.4 days, p = 0.0058), headache (– 3.1 days, p = 0.0222), chest congestion (– 2.8 days, p = 0.0386), shortness of breath (– 3.1 days, p = 0.0195) (Table 2). They also achieved the earliest recovery of smell (– 5.2 days, p = 0.0281) and ability to accomplish daily activities (– 4.6 days, p = 0.0118).

**Fig. 2:**
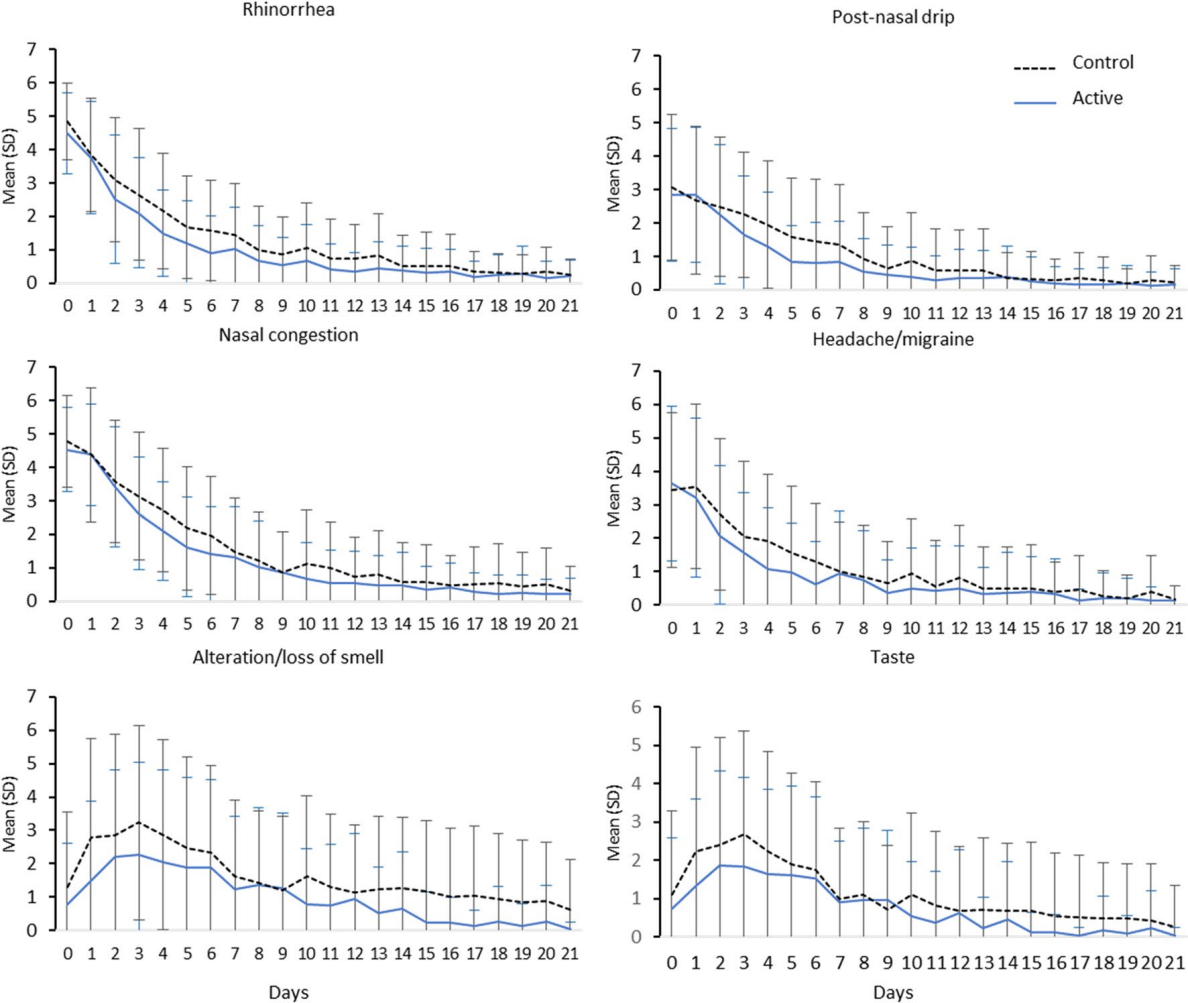
Time course of COVID-19 symptom intensity

Subjects with both nasal congestion and rhinorrhea also benefited from an earlier decrease in symptom intensity with nasal wash, especially for the most frequent and bothersome symptoms: rhinorrhea (p = 0.017 from Day 2 to Day 7), post-nasal drip (p = 0.025 from Day 3 to Day 7), plugged nose (p = 0.0352 from Day 3 to Day 6) and headache/migraine (p = 0.0217 from Day 2 to Day 6) (Fig. 2). Similarly, the intensity of smell disorders peaked at a lower intensity in active group versus control group (p = 0.0369 from Day 1 to Day 5) and this difference persisted by Day 21 (p = 0.0084 from Day 14 to Day 21). For taste disorders, similar while non-significant improvements were observed in the active group versus controls (p = 0.0522 from D1 to Day4 and p = 0.0914 from Day 13 to Day 21).

#### URTIs subjects

In all URTIs subjects, an earlier resolution of rhinorrhea (– 3.5 days, p = 0.0370), post-nasal drip (– 3.7 days, p = 0.0378), and overall sickness (– 4.3 days, p = 0.0248) was reported with nasal wash compared to control (Table 2). Subjects with severe rhinorrhea at baseline showed the earliest resolution of post-nasal drip (– 5.9 days, p = 0.0406) and cough (– 8.4 days, p = 0.0140). Subjects not taking concomitant medications presented the earliest resolution of rhinorrhea (– 4.4 days, p = 0.0375), nasal congestion (– 4.9 days, p = 0.0458) and overall sickness (– 5.5 days, p = 0.0138).

### Relief from nasal symptoms

In all subjects regardless of etiology, a significantly earlier relief of nasal symptoms was achieved in nasal wash group compared to controls for both nasal congestion (89.9% vs 71.9%, p < 0.001 by D3) and rhinorrhea (91.3% vs 74.9%, p < 0.001 by D3). From the first day of use, the majority of subjects from active group reported this relief as related to the device under investigation (nasal congestion: 71.4%; rhinorrhea: 73.0%).

### COVID-19 exacerbation

The switch from mild and moderate grade of COVID-19 infection to more severe one’s ^(27)^ was less reported in the active group compared to controls however the intergroup difference wasn’t significant (D7: 9.1% vs 13.7%, p = 0.7021; D14: 0% vs 12.8%, p = 0.07833; D21: 0% vs 7.9%, p = 0.5507). One subject in the control group had an evolution from “mild” to “severe” at the third week of follow-up, requiring breathing aid/oxygen therapy.

### SARS-CoV-2 transmission to household contacts

Of the 340 household members in the COVID-19 subgroup, 110 (32.7%) presented COVID-like/respiratory symptoms in the 15 days prior to the study.

**Fig. 3:**
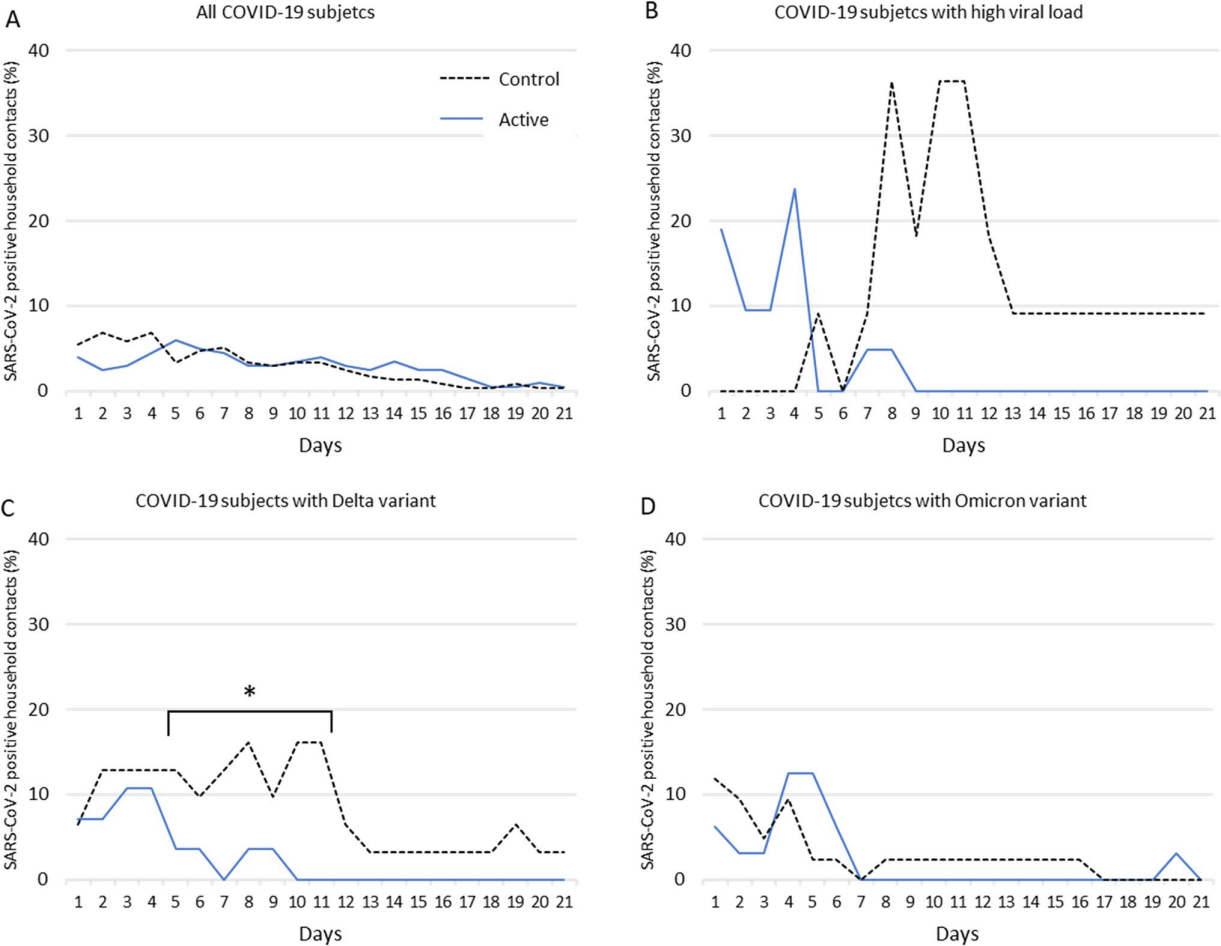
Percentage of SARS-CoV-2 positive household contacts from Day 1 to Day 21

The proportion of SARS-CoV-2 positive cases among household contacts from D1 to D21 ranged from 0.8 to 8.5% in the control group, and 0 to 9.0% in the active group, without significant intergroup differences (Fig. 3A). Among the subjects with higher viral load at baseline (≥ 5 log 10 copies/µL), the percentage of positive cases was significantly lower in the active group compared to the control group, ranging from 0 to 36.4% in the control group, and 0 to 23.8% in the active group (D10: p = 0.0168; D11: p = 0.0168) (Fig. 3B).

For the Delta variant subgroup, proportion of positive household contacts was lower in the active group (from 0 to 10.7%) than in controls (3.2–16.1%) (p-value = 0.0413, between D5 and D11) (Fig. 2C).

In the Omicron variant subgroup, the number of positive household contacts was similar in the active group (from 0 to 12.5%) and in controls (0–11.9%) with no significant intergroup differences (Fig. 3D).

### SARS-CoV-2 viral load

**Fig. 4:**
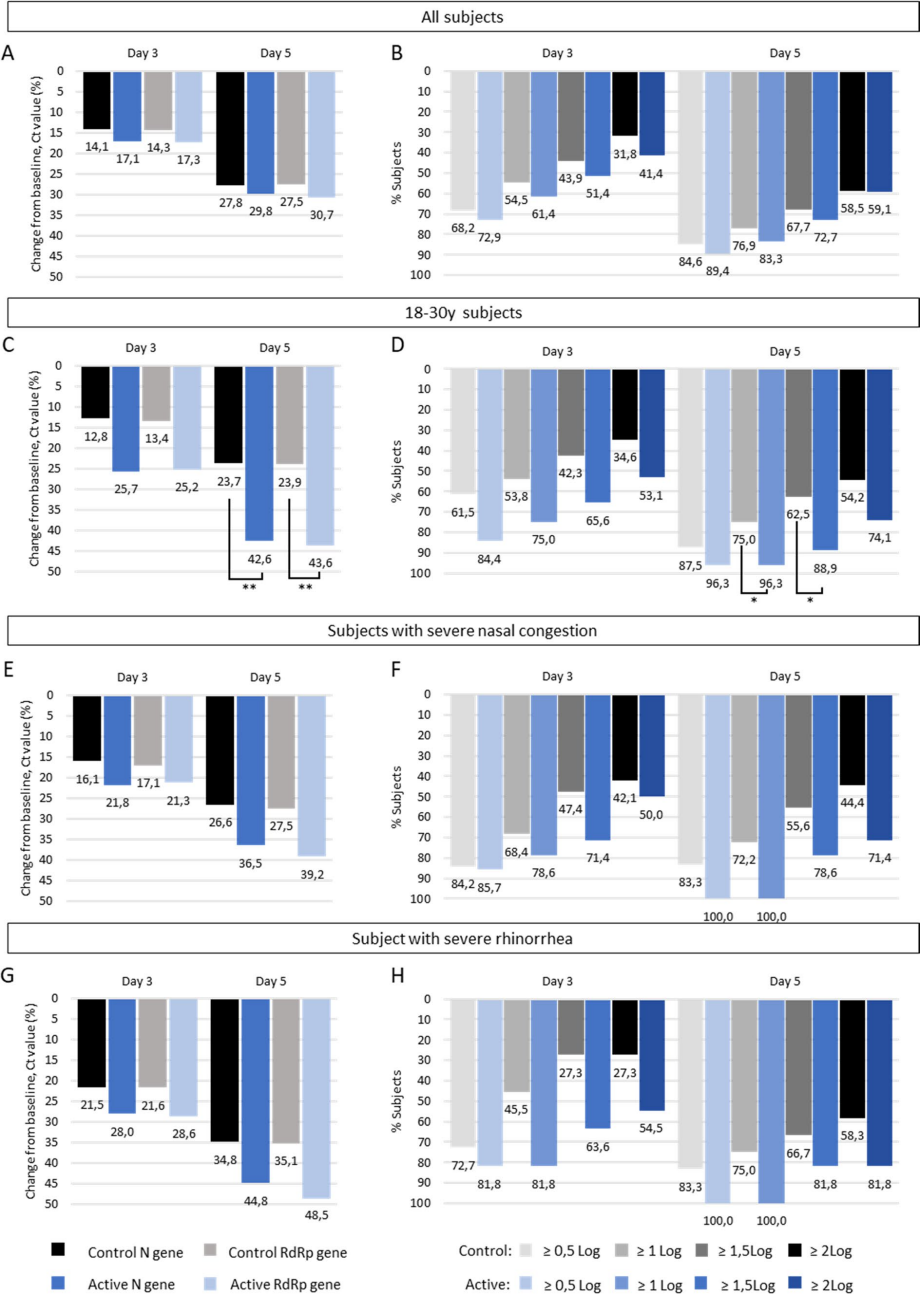
SARS-CoV-2 viral load

Baseline Ct values were similar in both groups, for N gene (26.3 ± 4.6 and 25.2 ± 4.6, p = 0.1026 in control and active groups respectively) and RdRp gene (26.6 ± 4.3 and 25.4 ± 4.6, p = 0.0551 in control and active groups respectively). Baseline ddPCR values were similar in both groups (5.89 ± 1.27 and 6.12 ± 1.37, p = 0.2526 in control and active groups respectively). Viral undetectability defined as a Ct value ≥ 42 was reached by D14 for the majority of subjects (D14: 86.5% and 83.1%; D21: 92.2% and 95.5% for control and active groups respectively). Changes in Ct values and Log_10_ copies/10,000 cells at D3 and D5 are shown in Fig. 4. Overall, earlier reductions in Ct values ​​and viral shedding were achieved from D3 in the nasal wash group compared to controls. By D5, higher changes in Ct values were achieved in the active group compared to controls for younger subjects (RdRp gene: – 43.6% vs – 23.9%, p = 0.0066) (Fig. 4C). There was also a trend in subjects with severe nasal congestion (RdRp gene: – 39.2% vs – 27.5%) and severe rhinorrhea (– 48.5% vs – 35.1%) without reaching statistical significance (p > 0.05) (Fig. 4E-G). Similar results were obtained for N gene.

By D5, a higher proportion of subjects in the active group achieved viral load falls ≥ 1 Log_10_ copies (96.3% vs 75.0% in active and control groups, p = 0.0420) and ≥ 1.5 Log_10_ copies (88.9% vs 62.5% in active and control groups, p = 0.0456) for younger subjects (18-30y, Fig. 4D). Similar reduction in viral shedding were reported among subjects with severe nasal congestion (change ≥ 1 Log_10_: 100% vs 72.2%; change ≥ 1.5 Log_10_: 78.6% vs 55.6% in active and control respectively). In subjects with severe rhinorrhea, the reduction in viral shedding with nasal wash was even more pronounced at D3, about twice that of the control group (change ≥ 1.5 Log_10_: 63.6% vs 27.3% in active and control respectively) while not reaching statistical significance.

### URTIs viral load

Baseline Ct values were similar in both groups (34.6 ± 4.6 and 31.5 ± 6.1 in control and active groups respectively). Viral undetectability was achieved by D14 in both groups for the majority of subjects (Supplementary Fig. 2).

A higher reduction of virus detectability was reached from day 3 in the active group compared to control group (– 62.1% vs – 36.4%). Subjects in the active group showed higher reduction of viral load, about twice that of the control group (D3: – 16.5% vs – 9.5%, p > 0.05; D5: – 25.4% vs – 12.1%, p = 0.0529 in active and control groups respectively).

### Compliance to nasal wash

The majority of subjects reported good compliance to nasal wash from D1 to D21 (i.e. uses/day ≥ 2 times, duration/use ≥ 1 s, performed in both nostrils) ranging from 65.0 to 86.4%. Ease of use of the nasal spray was reported by the majority of subjects throughout the study (82.6–86.6%).

### Overall tolerance

The majority of subjects (Min: 90.0%, Max: 98.6%) reported “good” to “excellent” overall tolerance to the device from D0 to D21.

### Safety: AEs, SAEs (All subjects)

Thirteen (3.4%) subjects had at least one adverse event (AE), with a total of 13 events (5 in the control group and 8 in the active group). Only 1 subject (0.3%) in the active group, had a non-serious event related to the device (nasal burning sensation), which resolved without sequelae on the same day. Two (0.5%) subjects had one severe adverse event (SAE), in the active group, a respiratory failure and a migraine, not linked with the medical device.

## Discussion

### Symptom resolution

Initiation of nasal wash with a seawater spray within 48 h of onset of upper respiratory symptom fostered significantly earlier improvement, relief and resolution of several nasal and respiratory symptoms in all subjects, regardless the etiology. While COVID-19 and URTI viral infections have a close semiology, they present some differences in terms of symptoms explaining why these improvements do not affect identical symptoms [35, 36].

In COVID-19 subjects, the earliest symptom resolution was observed when baseline nasal symptoms were upmost: notably for anosmia, post-nasal drip, face pain/heaviness, headache, dyspnea, chest congestion, sore throat and loss of appetite. They also recovered significantly sooner their ability to accomplish daily activities, being of interest since fatigue is the second most frequently reported symptom [37]. These results are consistent with the literature suggesting that nasal wash [19, 23] can foster earlier resolution of COVID-19 related symptoms. By promoting mechanical cleansing of mucus and inflammatory markers, seawater nasal wash may have facilitated odors access to the olfactory cleft [38, 39], probably explaining the earlier recovery of smell and taste observed in our study.

In URTI subjects, the use of seawater nasal wash fostered significantly earlier resolution of rhinorrhea, postnasal drip and overall sickness. Subjects with more severe baseline symptoms benefited from the soonest resolution of nasal congestion and cough. These findings are consistent with previous literature reporting earlier resolution of URTI symptoms (rhinorrhea, nasal congestion, cough) with nasal wash [8, 9, 12, 26, 32, 40].

### Viral load

An earlier reduction of SARS-CoV-2 viral load was reported with seawater nasal wash from day 3, among subjects with severe nasal symptoms and younger subjects. This reduction is consistent with previous studies with buffered normal saline [25] and hypertonic alkaline mineral-rich solution [37]. In contrast, a non-controlled study in hospitalized COVID-19 patients with a diluted seawater nasal spray didn’t reach significant viral load reduction at Day 3 and 5 [41]. In URTI subjects from active group, the reduction in viral load and viruses detectability reached twice that of control group by day 5. These results are consistent with previous study with hypertonic saline nasal washes and gargles to treat common cold [26]. Beyond the mechanical flushing effect of nasal wash, the use of undiluted seawater in our study may have enhanced mucociliary clearance, thus contributing to the earlier viral load decrease whereas viral infections usually impair mucociliairy clearance [17, 42].

### Household transmission

Households are high-risk setting for the transmission of SARS-CoV-2 via inhalation or contact with infected droplets [43, 44]. Therefore, concerns have been raised about the risk for nasal wash to increase SARS-CoV-2 transmission to household contacts. This study did not find any increase in SARS-CoV-2 positive cases among household contacts between subjects using nasal wash and controls. A lower percentage of SARS-CoV-2 positive cases was even reported among subjects with Delta variant and those with high viral load at D0. This absence of risk of household spread using nasal irrigation was also reported in the literature [24].

### Evolution of COVID-19 severity

In our study, evolution towards more severe COVID-19 was not increased and even less reported with nasal wash. These findings are consistent with the study from Baxter et al. reporting a reduced likelihood of hospitalization in high-risk COVID-19 patients using nasal irrigation [24]. Similarly, in the study from Yilmaz, no patient in the nasal wash group had to be hospitalized due to deterioration in their condition while 10% of the control group had to [37].

### Tolerance and safety

The majority of subjects regardless the etiology, reported good to excellent tolerance and very rare adverse events with seawater nasal wash. Similar favorable tolerance and safety profile has been reported in the literature [42, 45–47], with either isotonic saline [25, 32] or hypertonic mineral-rich solution buffered solutions [37]. On the opposite, side effects have been reported with solutions containing povidone iodine, or hydrogen peroxide [48, 49].

The current study presents several strengths. The prospective longitudinal design, the high number of subjects and the self-reported symptoms completed by virological assessments, reduced the possibility of biases. Moreover, the choice of a nasal wash method (nasal spray) widely used in URTIs, and the monitoring of the household environment made it closer to a real-life setting. Finally, the study population with different viruses and degrees of severity, allowed an assessment of nasal wash efficacy on a representative population suffering from upper respiratory tract symptoms.

Various limitations are present. First, the progress of the pandemic over time (variants, clinical presentations, waves, viral load kinetic) introduced some variability. Secondly, severe cases of COVID-19 were not included and about one third of COVID-19 subjects were vaccinated against SARS-CoV-2. Indeed, vaccination is usually associated with a reduced duration and intensity of symptoms [50]. Therefore, no extrapolation of our data is possible to severe COVID-19 patients.

## Conclusion

This study showed that undiluted seawater nasal wash can promote earlier relief, improvement, and resolution of upper respiratory tract related symptoms, limit household transmission, and decrease viral load in subjects with mild to moderate COVID-19 and URTIs infections.

### Supplementary Information

Below is the link to the electronic supplementary material.

Supplementary Table 1 (DOCX 16 KB)

Supplementary Figure 1: Symptom prevalence at baseline

Supplementary Figure 2: URTIs viral load

## Data Availability

Data available on request.

## Declarations

### Employment

GN is employee of Laboratoire de la Mer.
