# Supplementary material for: Seawater nasal wash to reduce symptom duration and viral load in COVID-19 and upper respiratory tract infections: a randomized controlled multicenter trial
Source: Eur Arch Otorhinolaryngol. 2024 Feb 20;281(7):3625–37. doi: 10.1007/s00405-024-08518-y (PMC11211132; doi:10.1007/s00405-024-08518-y)
Supplement: Supplementary file 1 — Supplementary Table 1 (DOCX 16 KB) [file 405_2024_8518_MOESM1_ESM.docx]

**Supplementary Table 1** Complete list of inclusion and exclusion criteria

| Inclusion Criteria | Exclusion Criteria |
| --- | --- |
| - Adult subjects (≥ 18 years) with self-reported nasal obstruction and/or rhinorrhea ≤48 hours due to COVID-19 or URTIs - Willing to have regular nasopharyngeal swabs as per protocol - Subjects agreeing to follow the study requirements during the whole study period - Subjects affiliated to social security - Subjects able to understand verbal and written local language and in capacity to fill-in questionnaire by himself | - Age over 65 years - Subjects requiring hospitalization - Subjects with severe COVID-19 symptoms - Inability or unwillingness to perform saline irrigations - subjects who have performed nasal wash in the previous week including the day of inclusion - Requirement to take regular medications administered by nasal route (topical treatment such as corticosteroids, antihistaminics, vasoconstrictors, inhalation) - Subjects intending to undergo nasal surgery during the study period or who underwent nasal surgery in the 3 previous months. - Pregnancy or breastfeeding - Cardiovascular pathologies: history of stroke, history of coronary artery disease, history of cardiac surgery, NYHA stage III or IV heart failure; - Asthma (requiring treatment) - Chronic pulmonary / respiratory pathology (cystic fibrosis obstructive pulmonary disease (COPD), pulmonary fibrosis) - Chronic renal disease (eg: renal failure) - Obesity (BMI ≥ 30) - Progressive cancer under treatment - Chronic haematological pathology - Chronic liver disease (eg: cirrhosis) - HIV infection / other immune deficiency (congenital or acquired immunosuppression) - Has received an organ or bone marrow transplant - Chronic neurological abnormality / disease - Immunosuppression - Subject having his 2nd injection of COVID-19 vaccine scheduled during the 3 weeks of study follow-up - Taking part in another interventional clinical trial or in the exclusion period to another study - Those who do not have access to email/internet - Not capable of giving informed consent - Hypersensitivity or known allergy to any component of the product - Patient with a member of his household already included in the study - Patient living in another region than the recruiting laboratory |
