# Supplementary figures and images for: Seawater nasal wash to reduce symptom duration and viral load in COVID-19 and upper respiratory tract infections: a randomized controlled multicenter trial
Source: Eur Arch Otorhinolaryngol. 2024 Feb 20;281(7):3625–37. doi: 10.1007/s00405-024-08518-y (PMC11211132; doi:10.1007/s00405-024-08518-y)

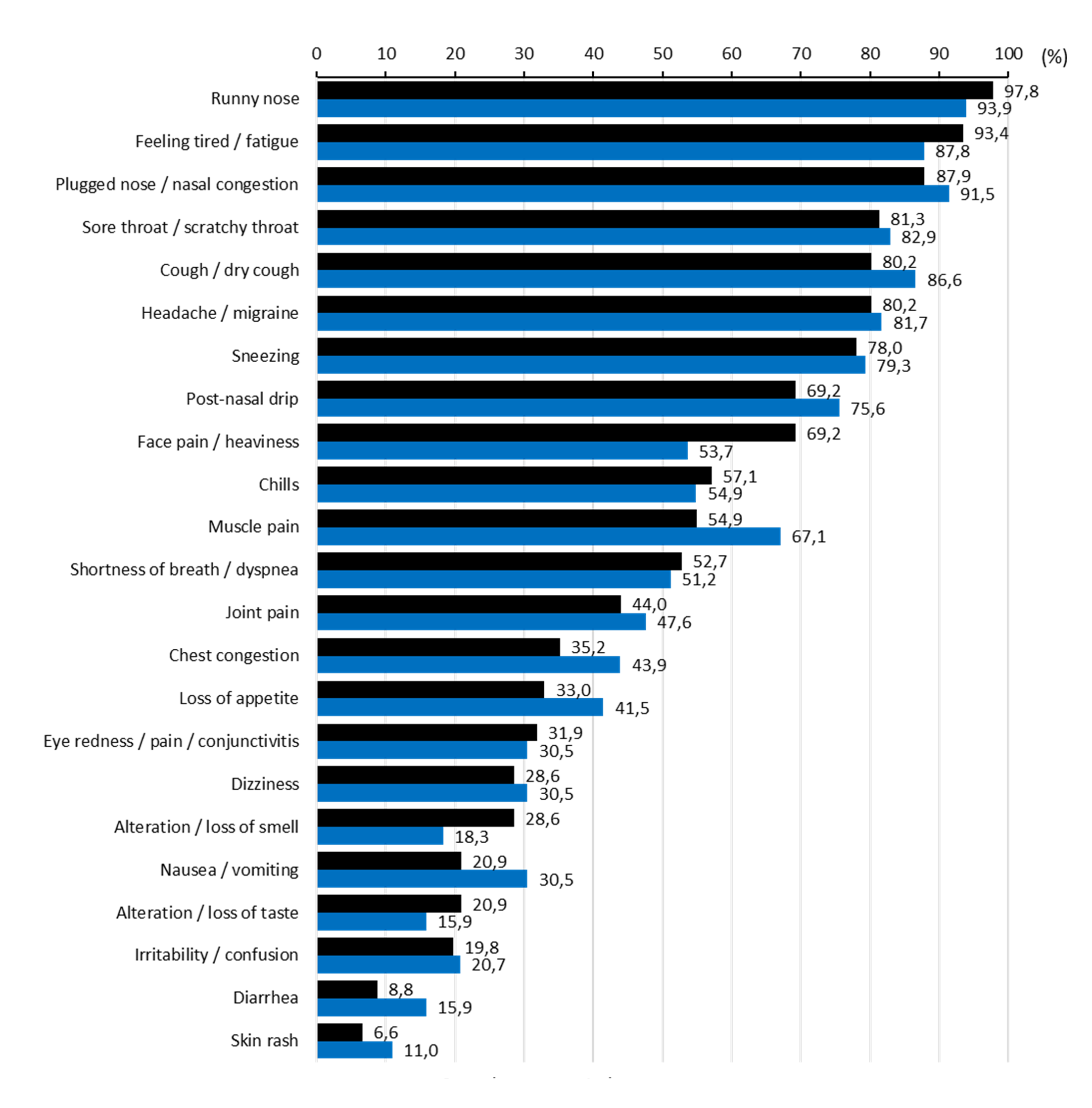

Supplement: Supplementary file 2 — Supplementary Figure 1: Symptom prevalence at baseline [file 405_2024_8518_MOESM2_ESM.tif]

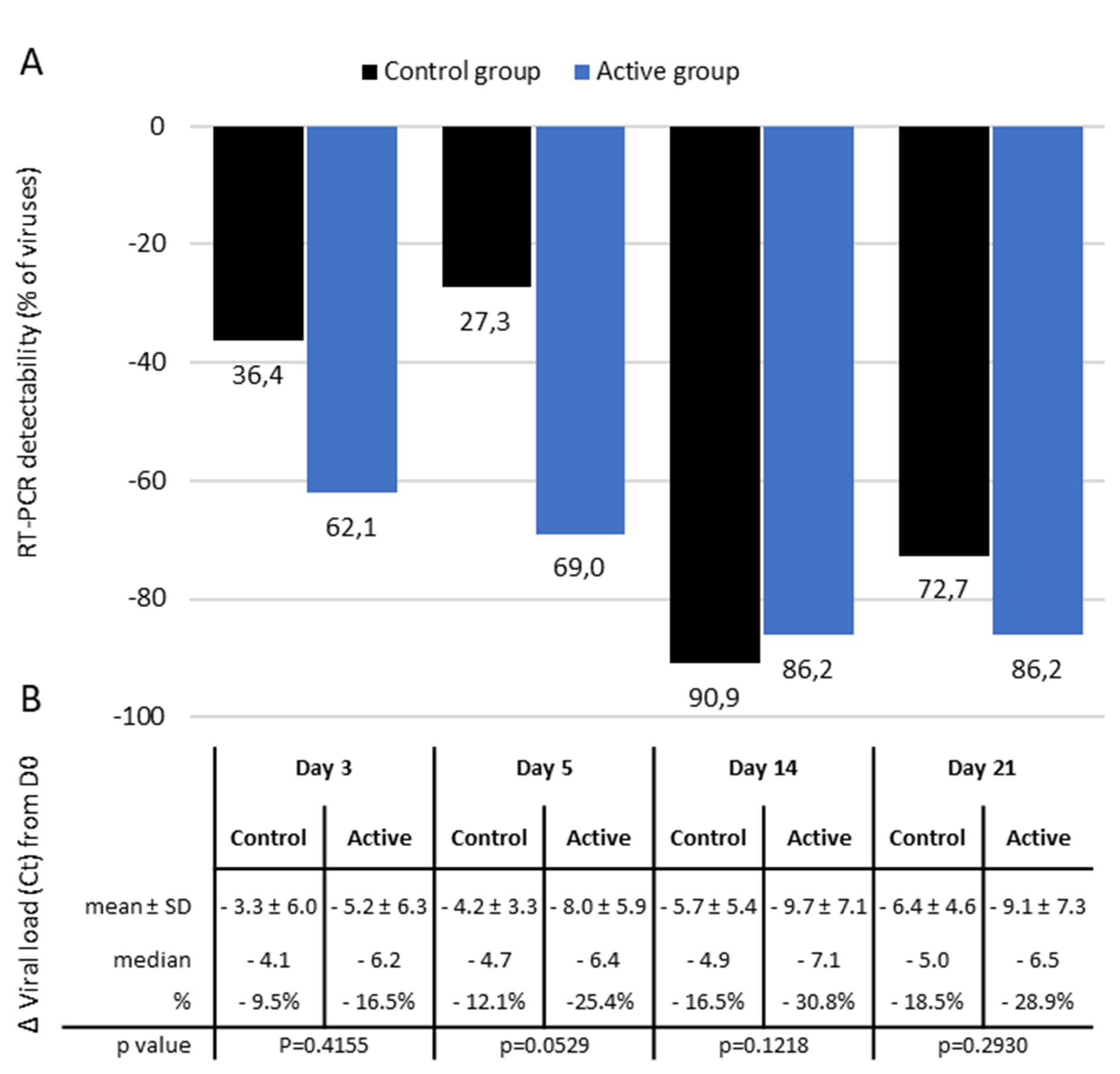

Supplement: Supplementary file 3 — Supplementary Figure 2: URTIs viral load [file 405_2024_8518_MOESM3_ESM.tif]
